# Effects of the Selective 5-HT7 Receptor Antagonist SB-269970 and Amisulpride on Ketamine-Induced Schizophrenia-like Deficits in Rats

**DOI:** 10.1371/journal.pone.0066695

**Published:** 2013-06-11

**Authors:** Agnieszka Nikiforuk, Tomasz Kos, Katarzyna Fijał, Małgorzata Hołuj, Dominik Rafa, Piotr Popik

**Affiliations:** Department of Behavioral Neuroscience and Drug Development, Institute of Pharmacology, Polish Academy of Sciences, Kraków, Poland; Chiba University Center for Forensic Mental Health, Japan

## Abstract

A wide body of evidence suggests that 5-HT7 receptors are implicated in a variety of central nervous system functions, including control of learning and memory processes. According to recent preclinical data, the selective blockade of these receptors may be a potential target for cognitive improvement in schizophrenia. The first aim of the present study was to evaluate the effects of the selective 5-HT7 receptor antagonist, SB-269970, and the antipsychotic drug with a high affinity for 5-HT7 receptors, amisulpride, on ketamine-induced deficits in attentional set-shifting and novel object recognition tasks in rats. Because the role of 5-HT7 receptor blockade in ameliorating positive and negative symptoms of schizophrenia remains equivocal, the second aim of these experiments was to examine the effectiveness of SB-269970 and amisulpride in reversing ketamine-induced deficits in prepulse inhibition of the startle reflex and in social interaction test in rats. The study revealed that acute administration of SB-269970 (1 mg/kg) or amisulpride (3 mg/kg) ameliorated ketamine-induced cognitive inflexibility and novel object recognition deficit in rats. Both compounds were also effective in attenuating ketamine-evoked disruption of social interactions. In contrast, neither SB-269970 nor amisulpride affected ketamine-disrupted prepulse inhibition or 50 kHz USVs accompanying social behaviour. In conclusion, antagonism of 5-HT7 receptors may represent a useful pharmacological approach in the treatment of cognitive deficits and some negative symptoms of schizophrenia.

## Introduction

A wide body of evidence supports a role for 5-HT7 (5-hydroxytryptamine 7, serotonin 7) receptors in the modulation of diverse processes of the central nervous system [1]–[3]. Several recent reports have suggested that these receptors may be involved in the control of learning and memory processes. While the involvement of 5-HT7 receptors in modulating cognitive processes under physiological conditions remains poorly understood [4], [5], recent data suggest that 5-HT7 antagonists are effective in overcoming cognitive impairments in an animal model of schizophrenia involving blockade of N-methyl-D-aspartate receptors (NMDARs) [6]–[9]. This issue is of special interest because several atypical antipsychotics, e.g., amisulpride and lurasidone, are characterised by a high affinity for 5-HT7 receptors [10], [11]. Interestingly, recent experimental data suggest that the antagonistic action of these compounds at 5-HT7 receptors may account for their procognitive effects in models of schizophrenia-like cognitive deficits [7]

In addition to positive and negative symptoms, cognitive deficits are increasingly recognised as a core feature of schizophrenia [12]. Several cognitive domains were identified as commonly deficient in schizophrenic patients [13]. Some of these deficits can be assessed in animal models in a way that is analogous to human tests [14], [15]. Specifically, cognitive flexibility, a component of executive control subserved by the prefrontal cortex (PFC), can be evaluated in rodents in the attentional set-shifting task (ASST) [16]. In this paradigm, rats must select a bowl containing a food reward based on the ability to discriminate the odours and the media covering the bait. The ASST requires rats to initially learn a rule and to form an attentional “set” within the same stimulus dimensions. At the extra-dimensional (ED) shift stage, the essential phase of the task, animals must switch their attention to a new, previously irrelevant stimulus dimension and, for example, discriminate only between the odours and no longer between the media covering the bait. The animals' performance at the ED stage is regarded as an index of cognitive flexibility.

The novel object recognition (NOR) test for rodents has been increasingly used as an ethologically relevant paradigm for studying visual episodic memory [17]. This task is based on spontaneous exploration of novel and familiar objects. Successful object recognition is demonstrated by a longer time spent interacting with the novel object in the retention trial. Therefore, the ASST and NOR task provide a useful translational approach in studying abnormalities relevant to schizophrenia.

Noncompetitive antagonists of the NMDAR, such as ketamine and phencyclidine (PCP), produce a behavioural syndrome in healthy humans that closely resembles the symptoms of schizophrenia [18]. Therefore, NMDAR-based models are commonly used to mimic a schizophrenia-like state in laboratory animals [19]. Acute administration of NMDAR antagonists evokes a broad range of schizophrenia-like symptoms, including cognitive deficits. Because ketamine is commonly used in the clinic to model the transient neurocognitive impairments in healthy volunteers [20], the ketamine-based animal model may represent a valuable tool in preclinical research.

Therefore, the first aim of the present study was to evaluate the effects of the selective and high affinity (p*K*(_i_)  =  8.9) 5-HT7 receptor antagonist, SB-269970 [21], and the antipsychotic drug with a high affinity (K_i_  =  11.5 nM) for 5-HT7 receptors, amisulpride [10], on ketamine-induced deficits in attentional set-shifting and novel object recognition tasks in rats.

In contrast to the beneficial action of 5-HT7 antagonists on schizophrenia-like cognitive impairments, the role of 5-HT7 receptor blockade in ameliorating positive and negative symptoms remains equivocal [22]. Because prominent deficits in sensorimotor gating and social withdrawal are observed in schizophrenia patients, the second aim of this study was to examine the effectiveness of SB-269970 and amisulpride in reversing ketamine-induced deficits in prepulse inhibition (PPI) of the startle reflex, an operational measure of sensorimotor gating, and in social interaction (SI) in rats. Because social behaviour is accompanied by ultrasonic vocalisations (USVs) [23], [24], we also used this index as a readout of negative symptoms, i.e., social withdrawal.

## Materials and Methods

### Animals

The experiments were conducted in accordance with the NIH Guide for the Care and Use of Laboratory Animals and were approved by the Ethics Committee for Animal Experiments, Institute of Pharmacology.

Male Sprague-Dawley rats (Charles River, Germany), weighing 200–250 g (ASST, NOR and PPI tests) or 125–150 g (SI test) on arrival, were housed in a temperature-controlled (21±1°C) and humidity-controlled (40–50%) colony room under a 12/12 h light/dark cycle (lights on at 06:00 h). For NOR and PPI studies, rats were group-housed (5 rats/cage) with free access to food and water. For the ASST, rats were individually housed with a mild food restriction (15 g of food pellets per day) for at least one week prior to testing. For the SI, rats were individually housed for 5 days before the start of the procedure with free access to food and water. Behavioural testing was performed during the light phase of the light/dark cycle.

### Attentional set-shifting task

#### Apparatus

Testing was conducted in a Plexiglas apparatus (length x width x height: 38×38×17 cm) with the grid floor and wall dividing half of the length of the cage into two sections. During testing, one ceramic digging pot (internal diameter of 10.5 cm and a depth of 4 cm) was placed in each section. Each pot was defined by a pair of cues along with two stimulus dimensions. To mark each pot with a distinct odour, 5 μl of a flavouring essence (Dr. Oetker®, Poland) was applied to a piece of blotting paper fixed to the external rim of the pot immediately prior to use. A different pot was used for each combination of digging medium and odour; only one odour was ever applied to a given pot. The bait (one-third of a Honey Nut Cheerio, Nestle®) was placed at the bottom of the “positive” pot and buried in the digging medium. A small amount of powdered Cheerio was added to the digging media to prevent the rat from trying to detect the buried reward by its smell.

#### Procedure

The procedure was adapted from Birrell and Brown [16] and lasted 3 days for each rat.

Day 1, habituation: rats were habituated to the testing area and trained to dig in the pots filled with sawdust to retrieve the food reward. Rats were transported from the housing facility to the testing room where they were presented with one unscented pot (filled with several pieces of Cheerios) in their home cages. After the rats had eaten the Cheerio from the home cage pot, they were placed in the apparatus and given three trials to retrieve the reward from both of the sawdust-filled baited pots. With each exposure, the bait was covered with an increasing amount of sawdust.

Day 2, training: rats were trained on a series of simple discriminations (SD) to a criterion of six consecutive correct trials. For these trials, rats had to learn to associate the food reward with an odour cue (e.g., arrack vs. orange, both pots filled with sawdust) and/or a digging medium (e.g., plastic balls vs. pebbles, no odour). All rats were trained using the same pairs of stimuli. The positive and negative cues for each rat were presented randomly and equally. These training stimuli were not used again in later testing trials.

Day 3, testing: rats performed a series of discriminations in a single test session. The first four trials at the beginning of each discrimination phase were a discovery period (not included in the six criterion trials). In subsequent trials, an incorrect choice was recorded as an error. Digging was defined as any distinct displacement of the digging media with either the paw or the nose; the rat could investigate a digging pot by sniffing or touching without displacing material. Testing was continued at each phase until the rat reached the criterion of six consecutive correct trials, after which testing proceeded to the next phase.

In the simple discrimination involving only one stimulus dimension, the pots differed along one of two dimensions (e.g., digging medium). For the compound discrimination (CD), the second (irrelevant) dimension (i.e., odour) was introduced but the correct and incorrect exemplars of the relevant dimension remained constant. For the reversal of this discrimination (Rev 1), the exemplars and relevant dimension were unchanged but the previously correct exemplar was now incorrect and vice versa. The intra-dimensional (ID) shift was then presented, consisting of new exemplars of both the relevant and irrelevant dimensions with the relevant dimension remaining the same as previously. The ID discrimination was then reversed (Rev 2) so that the formerly positive exemplar became the negative one. For the extra-dimensional (ED) shift, a new pair of exemplars was again introduced, but this time a relevant dimension was also changed. Finally, the last phase was the reversal (Rev 3) of the ED discrimination. The exemplars were always presented in pairs and varied so that only one animal within each treatment group received the same combination. The assignment of each exemplar in a pair as being positive or negative at a given phase and the left-right positioning of the pots in the test apparatus on each trial were randomised. Table 1 outlines the progressions through ASST phases including exemplars of odours and media used and their assignment into pairs.

**Table 1:** Order of discriminations performed.

Phase	Relevant Dimension	Discrimination	
**SD**	**medium**	**clay pellets**	silk
**CD**	**medium**	**clay pellets**	silk
		rum	cream
**Rev 1**	**medium**	clay pellets	**silk**
		rum	cream
**ID**	**medium**	**shredded paper**	metallic filler
		spicy	vanilla
**Rev 2**	**medium**	shredded paper	**metallic filler**
		spicy	vanilla
**ED**	**odour**	**lemon**	almond
		cotton wool	crumpled tissue
**Rev 3**	**odour**	lemon	**almond**
		cotton wool	crumpled tissue

An example of the cue combinations used in the attentional set shifting task (ASST) of rats that were shifted from the digging medium to odour as the relevant dimension. Rats performed a series of 7 discriminations: simple discrimination (SD), compound discrimination (CD), reversal 1 (Rev1), intradimensional shift (ID), reversal 2 (Rev 2), extradimensional shift (ED), reversal 3 (Rev 3). The correct exemplar (shown in bold) was paired with either of two exemplars from the irrelevant dimension (i.e., at the CD phase, the clay pellets were paired with either rum or cream odour, etc). In the ID and ED, there were novel pairs of exemplars of each dimension.

### Novel object recognition task

#### Apparatus

Rats were tested in a dimly lit (25 Lux) open field made of dull grey plastic (length x width x height: 66×56×30 cm). After each measurement, the floor was cleaned and dried.

#### Procedure

Rats were habituated to the arena (without any objects) for 5 min 24 h before testing [17]. The test consisted of two 3-min trials separated by an inter-trial interval (ITI) of 1 h. During the first trial (familiarisation, T1), two identical objects (A1 and A2) were presented in opposite corners, approximately 10 cm from the walls of the open field. In the second trial (retention, T2), one of the objects was replaced by a novel one (A = familiar and B = novel). Animals were returned to their home cage after T1. The objects used were a glass bulb filled with gravel and a plastic bottle filled with sand. The height of the objects was comparable (∼12 cm), and they were heavy enough not to be displaced by the animals. Half of the animals from each group received the glass bulb as a novel object and half the plastic bottle. The location of the novel object in the recognition trial was randomly assigned for each rat. Exploration of an object was defined by looking, licking, sniffing or touching the object while sniffing, but not leaning against, standing or sitting on the object. Any rat spending less than 5 s exploring the two objects within 3 min of T1 or T2 was eliminated from the study. The exploration time of the objects and the distance travelled were measured using the Any-maze® tracking system. Based on the exploration time (E) of the two objects, a discrimination index was calculated by DI  =  (E_B_–E_A_)/(E_A_+E_B_).

### Social interaction test

#### Apparatus

The experiments were conducted in the open field arena (length x width x height: 57×67×30 cm) made of black Plexiglas. The arena was dimly illuminated with an indirect light of 18 Lux. The behaviour of the rats was recorded by two cameras placed above the arena and connected to the Noldus MPEG recorder 2.1. Videos were analysed off-line by the Noldus The Observer® XT, version 10.5. In addition, we measured the number of ultrasonic calls using an ultrasound heterodyne bat detector microphone (Ultrasound Advice, UK) set to record the high-frequency ∼ 50 kHz vocalisations. Following hardware transformation into the audible range, the recordings were automatically added as the “soundtracks” into the mpeg films recorded by the computer and manually counted off-line.

#### Procedure

Rats were individually housed for 5 days prior to the start of the procedure. On the fifth day of social isolation, all rats were transferred to the experimental room and individually adapted to the open field arena for 7 min. Afterward, the rats were handled, weighed and half were dyed with a gentian violet (2% Methylrosanilinium chloride) on the rear part of the body. On the test day (the sixth day of social isolation), two unfamiliar rats of matched body weight (±5 g), one white and one dyed, were placed in the open field arena and their behaviour was recorded for 10 min. Both rats in a given pair received the same treatment. Social interaction time was measured for each rat separately and expressed as a summed score per each pair of rats. The following active social behaviours were scored: sniffing (rat sniffs the conspecific's parts of the body, including the anogenital region), social grooming (rat licks and chews the fur of the conspecific), following (rat moves towards and follows the other rat), mounting (rat stands on the conspecific’s back) and climbing (rat climbs over the conspecific‘s back).

### Prepulse inhibition of the acoustic startle response

#### Apparatus

Rats were tested in the startle apparatus (Med Associates, St. Albans, USA), consisting of acrylic animal holders with a grid floor made of stainless bars and mounted onto a startle platform placed in a ventilated, sound-attenuating chamber. Acoustic stimuli were generated by two speakers, a background noise speaker and a stimulus speaker, placed at the back of the chamber 7 cm from an animal holder. Startle responses were detected and transduced by the load cell and then digitised and stored by Startle Reflex software (Med Associates, version 5).

#### Procedure

Sessions started with a 5-min acclimatisation period. A 62-dB background white noise was continuously presented once animals were placed in the test chambers and was maintained throughout the whole session. The session was arranged into 3 blocks. First, the animals were exposed to 10 pulse-alone trials included to induce habituation to startle. These 10 pulse trials were followed by a series consisting of pulse-alone trials [intensity: 120 dB, duration: 40 ms, (P)], trials of pulse preceded by either 70, 73 or 76 dB prepulses [duration: 20 ms, (PP)] applied 100 ms before pulse (P), prepulses alone [intensity 70, 73 and 76 dB, duration 20 ms] and no-stimulus trials, presented in a random order (second block). The inter-trial interval was 20 seconds. The third block consisted of 10 pulse-alone trials. The session had a total of 48 trials.

For calculations, the measures obtained in the second block were used. The mean response amplitude for pulse alone [P] and prepulse+pulse [PP] trials was computed for each rat, and the PPI was determined according to the formula PPI (%)  =  [(P-PP)/P]*100.

### Drugs

Ketamine (aqueous solution (115.34 mg/ml), Vetoquinol Biowet, Gorzów Wielkoposki, Poland) was diluted in distilled water to the appropriate concentration. SB-269970 hydrochloride (Tocris, Bristol, UK) was dissolved in distilled water. Amisulpride was dissolved in distilled water with a drop of acetic acid, and the solution was neutralised with 0.1 N NaOH. Clozapine (Ascent Scientific, Bristol, UK) was dissolved in 0.1 N HCl supplemented with distilled water to the appropriate volume. Drugs or vehicle (physiological saline) were administered in a volume of 1 ml/kg of body weight.

### Drug administration

#### ASST

Ketamine (0 or 10 mg/kg, SC) was administered 75 min before the task, and SB-269970 (0, 0.3 or 1 mg/kg, IP) or amisulpride (0 or 3 mg/kg, IP) was administered 15 min before the ketamine (or vehicle) injection.

#### NOR

Ketamine at a dose of 20 mg/kg (IP) or vehicle was administered 45 min before the acquisition trial (T1), and SB-269970 (0, 0.3 or 1 mg/kg, IP) or amisulpride (0 or 3 mg/kg, IP) was given 30 min before the ketamine (or vehicle) injection.

#### SI

Ketamine at a dose of 20 mg/kg (IP) was given 30 min before the test. SB-269970 (0.3 or 1 mg/kg, IP) or amisulpride (3 mg/kg, IP) was given 30 min before the ketamine injections.

#### PPI

Ketamine at a dose of 10 mg/kg or vehicle was administered subcutaneously (SC) 5 min before the start of the test session. SB-269970 (0, 0.3 or 1 mg/kg, IP), amisulpride (0 or 3 mg/kg, IP) or clozapine (0 or 5 mg/kg, IP) was administered 10 min before the injection of ketamine (or vehicle).

Doses of ketamine and schedules of administration, adopted from published protocols [25] and preliminary experiments, have been demonstrated to produce a reliable impairment in the ASST, NOR, PPI and SI test. Data from this laboratory showed that the ketamine-induced deficits in set-shifting and object recognition were reversed by sertindole, demonstrating the predictive validity of these assays [25].

The pre-treatment time of ketamine in the ASST (75 min) was longer than in other tests. However, ketamine administered at shorter pre-treatment times would preclude rats from performing the task due to motivational and motor effects (unpublished observations). Moreover, using a 75-min pre-treatment schedule, we were able to detect a selective impairment in the ED stage [25]. Similarly, Gastambide et al. [26] recently demonstrated that deficits in cognitive flexibility could be measured up to 4 h following ketamine or PCP administration. Thus, extended schedules of ketamine administration allowed for demonstrating specific impairments of higher order cognitive functions in the absence of confounding motivational and motor effects.

Doses of SB-269970 and amisulpride were based on a previous study [7] demonstrating their efficacy in reversing the PCP-induced impairment in the NOR task. The dose of clozapine was determined by the ability to reverse PPI impairments in various pharmacological models on the basis of published work [27] and preliminary studies conducted in our laboratory.

### Statistical analysis

#### ASST

The number of trials required to achieve the criterion of 6 consecutive correct responses was recorded for each rat and for each discrimination phase of the ASST. Data were analysed using three-way mixed-design ANOVAs with ketamine and respective drug treatments as between-subject factors and discrimination phase as a repeated measure.

#### NOR

Data on exploratory preference were analysed using three-way mixed-design ANOVAs with ketamine and the respective drug treatment as between-subject factors and object as a repeated measure; DI data were analysed by two-way ANOVAs, and distance travelled was analysed using mixed-design ANOVAs with ketamine and the respective drug treatment as between-subject factors and trial as a repeated measure.

#### SI

Total time of social interaction and the number of ultrasonic calls were analysed by one-way ANOVAs.

#### PPI

Percentage PPI data were analysed using two-way ANOVAs with two between-subject factors (ketamine and the respective drug treatment) and prepulse intensity as a within-subject factor. Pulse amplitude values were subjected to two-way ANOVAs.

Post hoc comparisons were performed using Newman-Keuls tests. Statistical analyses were performed with the use of Statistica 10.0 for Windows. The alpha value was set at p<0.05.

## Results

### Attentional set-shifting task

**Figure 1:**
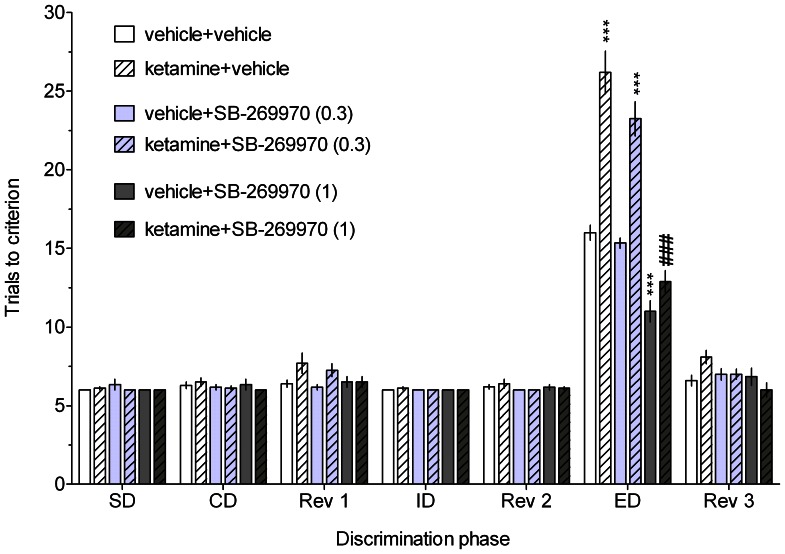
The effect of SB-269970 on the ketamine-induced cognitive impairment in the ASST. Results represent the mean ± S.E.M. number of trials required to reach the criterion of 6 consecutive correct trials for each of the discrimination phases. N = 8–10 rats per group. Symbols: ***p<0.001 vs. ED performance in the vehicle-treated group; ^###^p<0.001 vs. ED performance in the ketamine-treated group.

**Figure 2:**
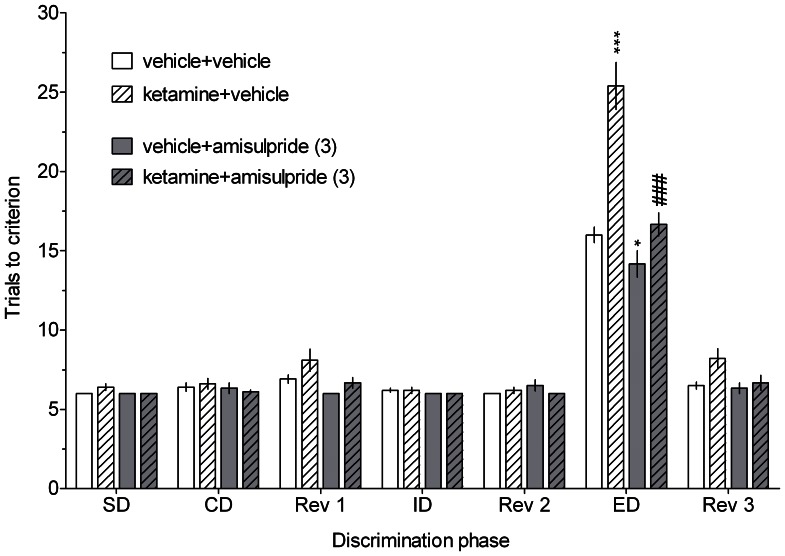
The effect of amisulpride on the ketamine-induced cognitive impairment in the ASST. Results represent the mean ± S.E.M. number of trials required to reach the criterion of 6 consecutive correct trials for each of the discrimination phases. N = 9–10 rats per group. Symbols: ***p<0.001, *p<0.05 vs. ED performance in the vehicle-treated group, ^###^p<0.001 vs. ED performance in the ketamine-treated group.

Acute administration of ketamine significantly and specifically impaired rats’ performance in the ED stage of the ASST, as indicated by an increased number of trials to criterion during this phase (Figures 1 and 2). There was no significant drug effect during any other discrimination stage.

Administration of SB-269970 (1 but not 0.3 mg/kg; Figure 1) or amisulpride (3 mg/kg, Figure 2) ameliorated the ketamine-induced deficit and promoted cognitive flexibility in control rats.

Three-way mixed-design ANOVAs revealed significant interactions between discrimination phase, ketamine and respective drug treatment: F[12,264] = 6.31, p<0.001 (SB-269970, Figure 1) and F[12,156] = 6.64, p<0.001 (amisulpride, Figure 2).

### Novel object recognition task

**Figure 3:**
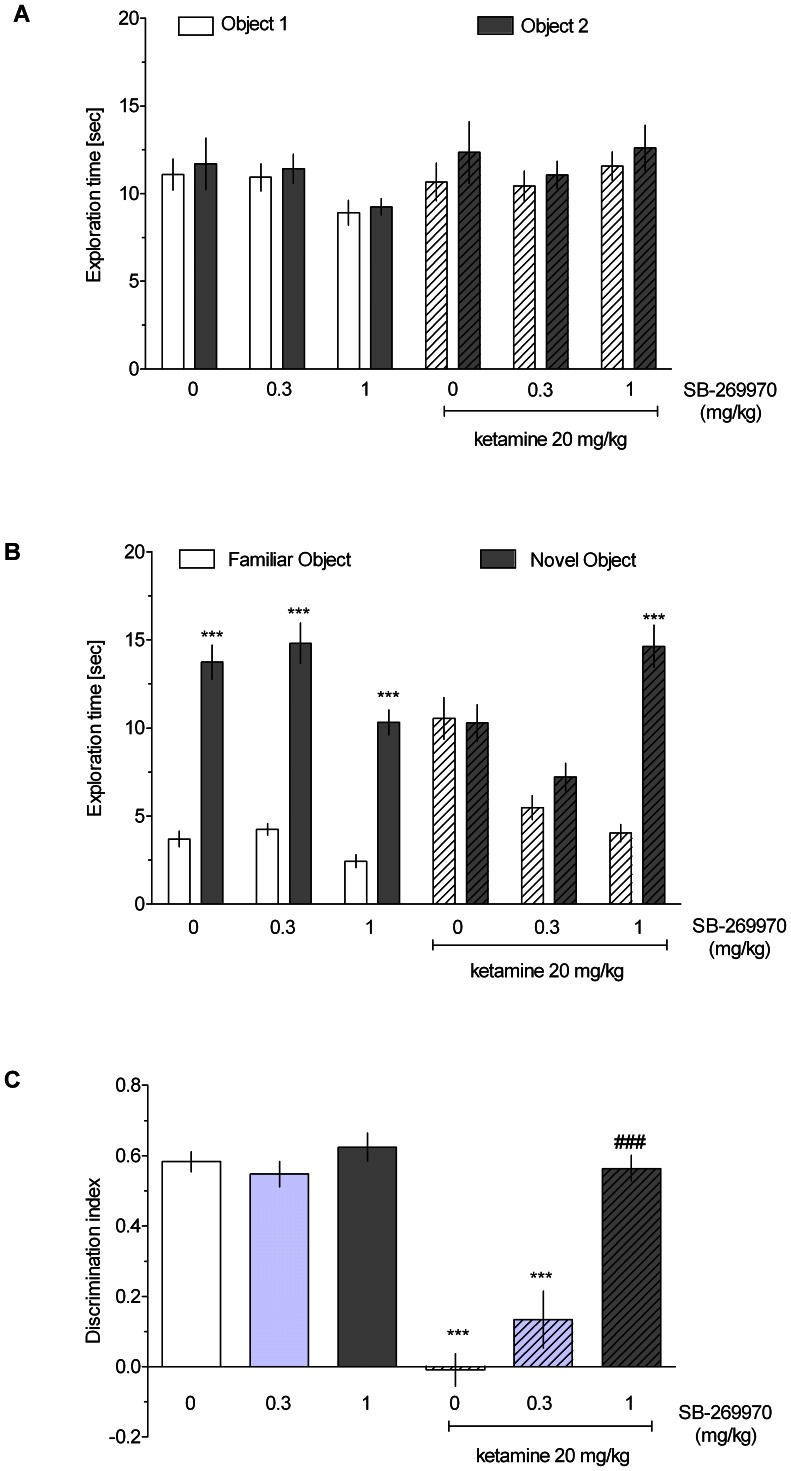
The effect of SB-269970 on ketamine-induced cognitive impairment in the NOR test. Data are shown as the mean ± S.E.M. N* = *8–9 rats per group. **A** Exploration time of two identical objects in the acquisition trial (T1); **B** Exploration time of a novel and a familiar object in the retention trial (T2). Symbols: ***p<0.001 significant difference in time spent exploring the novel compared with the familiar object; **C** Discrimination Index (DI). Symbols: ***p<0.001 significant reduction in DI compared to the vehicle-treated group, ^###^p<0.001, significant improvement in DI compared to the ketamine-treated group.

**Figure 4:**
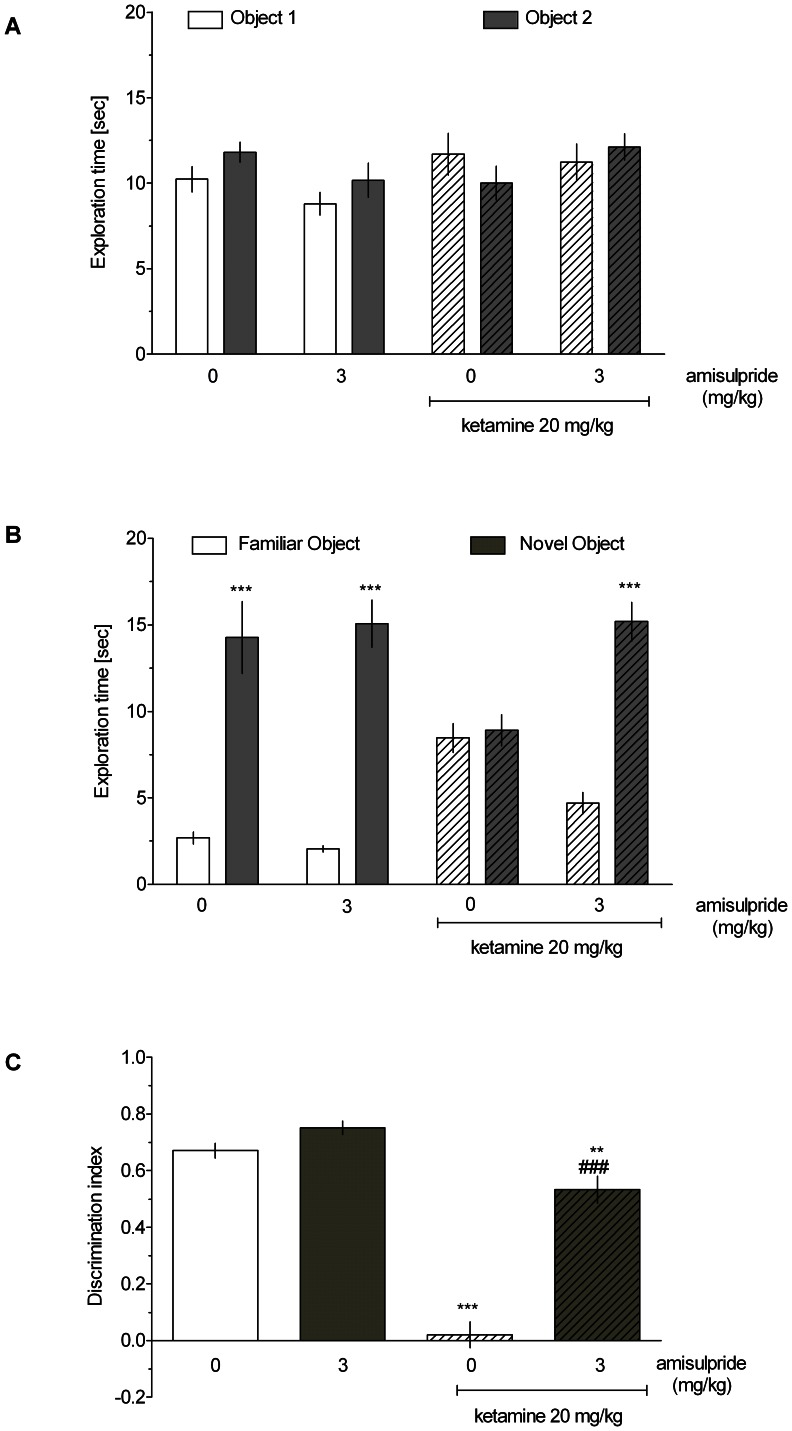
The effect of amisulpride on ketamine-induced cognitive impairment in the NOR test. Data are shown as the mean ± S.E.M. N = 9–10 rats per group. **A** Exploration time of two identical objects in the acquisition trial (T1); **B** Exploration time of a novel and a familiar object in the retention trial (T2). Symbols: ***p<0.001 significant difference in time spent exploring the novel compared with the familiar object; **C** Discrimination index (DI). Symbols: ***p<0.001, **p<0.01, significant reduction in DI compared to the vehicle-treated group, ^###^p<0.001, significant improvement in DI compared to the ketamine-treated group.

As demonstrated in Figures 3A and 4A, there were no significant differences in the time spent exploring two identical objects in the acquisition phase in any group (three-way ANOVA interactions for SB-269970 and amisulpride experiments: F[2], [47] = 0.07, NS and F[1], [35] = 1.59, NS, respectively). In the retention trial, vehicle-treated rats, but not ketamine-treated rats, spent significantly more time exploring the novel object compared with the familiar one (Figures 3B and 4B, three-way ANOVA interactions for SB-269970 and amisulpride experiments: F[2], [47] = 31.53, p<0.001 and F[1], [35] = 10.38, p<0.001, respectively). Thus, administration of ketamine abolished the ability to discriminate the novel and familiar objects. This deficit was reduced by SB-269970 (1 mg/kg, Figure 3B) and amisulpride (3 mg/kg, Figure 4B). Moreover, two-way ANOVAs revealed significant ketamine x drug treatment interactions on DI measures: F[2], [47] = 16.02, p<0.001 (SB-269970, Figure 3c) and F[1], [35] = 34.36, p<0.001 (amisulpride, Figure 4c). Post hoc analyses revealed that SB-269970 (1 mg/kg, Figure 3C) and amisulpride (3 mg/kg, Figure 4C) significantly attenuated the ketamine-induced DI reduction. However, amisulpride only partially ameliorated the ketamine-evoked impairment, as there was still a significant difference between the amisulpride+ketamine- and vehicle-treated groups.

Treatment effects on distance travelled by rats in familiarisation and retention trials are shown in Table 2. There were no significant two-way ANOVA interactions with either SB-269970 (F[2], [47] = 0.41, NS) or amisulpride treatment (F[1], [35] = 0.96, NS).

**Table 2:** Distance travelled in the familiarisation (T1) and retention (T2) trials.

Treatment (mg/kg)	T1 (m)			T2 (m)		
vehicle+vehicle	12.4	±	1.6	9.2	±	1.5
vehicle+SB-269970 (0.3)	12.4	±	0.8	8.3	±	0.6
vehicle+SB-269970 (1)	12.4	±	0.8	7.2	±	1.1
ketamine+vehicle	11.1	±	0.8	7.9	±	1.0
ketamine+SB-269970 (0.3)	10.3	±	1.3	6.4	±	0.8
ketamine+SB-269970 (1)	14.7	±	0.7	10.9	±	1.1
vehicle+vehicle	12.4	±	1.1	10.1	±	1.2
vehicle+amisulpride (3)	12.1	±	0.7	8.3	±	0.7
ketamine+vehicle	13.6	±	1.1	8.9	±	0.9
ketamine+amisulpride (3)	14.1	±	0.7	9.9	±	0.8

### Social interaction test

**Figure 5:**
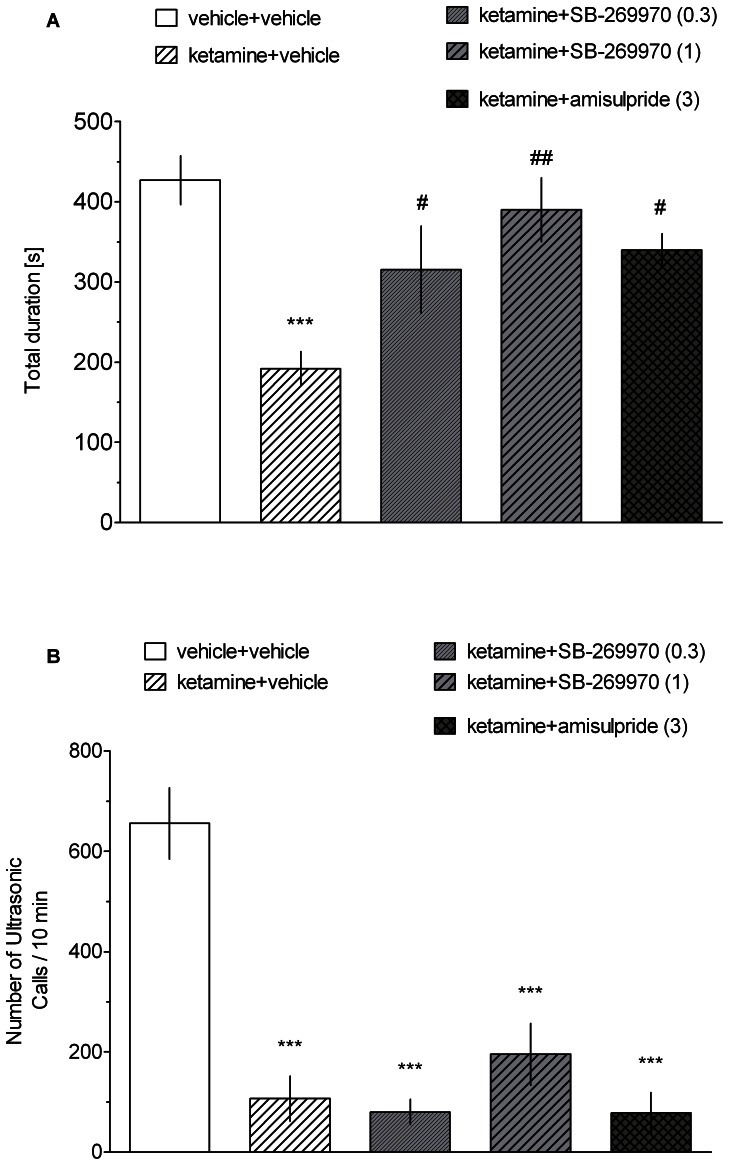
The effect of SB-269970 and amisulpride on the ketamine-disrupted social behaviour and ultrasonic vocalisations. Results represent the mean ± S.E.M. of total time spent in active social interaction (A) and the total number of USVs (B) per pair of rats. N = 5–6 pairs of rats per group. Symbols: ***p<0.001 significant reduction compared to the vehicle-treated group, ^##^p<0.01, ^#^p<0.05 significant reversal of ketamine-induced deficit.

As demonstrated in Figure 5A, administration of ketamine caused a significant reduction in the total social interaction time compared to the vehicle-treated animals (one-way ANOVA: F[4], [21] = 7.72, p<0.001). SB-269970 (0.3 and 1 mg/kg, p<0.05 and p<0.01, respectively) and amisulpride (3 mg/kg, p<0.05) ameliorated the ketamine-induced decrease in social interaction. Neither SB-269970 (0.3 and 1 mg/kg) nor amisulpride (3 mg/kg) affected the social behaviour of control rats (total time of SI: 408±25 s and 440±18 s for SB-269970 and amisulpride, respectively; data not shown). Figure 5B shows the number of ultrasonic vocalisations produced by the rats during the social encounter. One-way ANOVA demonstrated significant effects of treatment (F[4], [21] = 22.823, p<0.001), and subsequent post-hoc tests revealed that ketamine treatment reduced the number of calls. This effect of ketamine was not counteracted by either SB-269970 or by amisulpride; both compounds, however, reduced the number of calls when given with vehicle (mean number of calls 148.6±32.92 and 346±105, respectively) compared to vehicle (656±13); data not shown.

### Prepulse inhibition of the acoustic startle response

**Figure 6:**
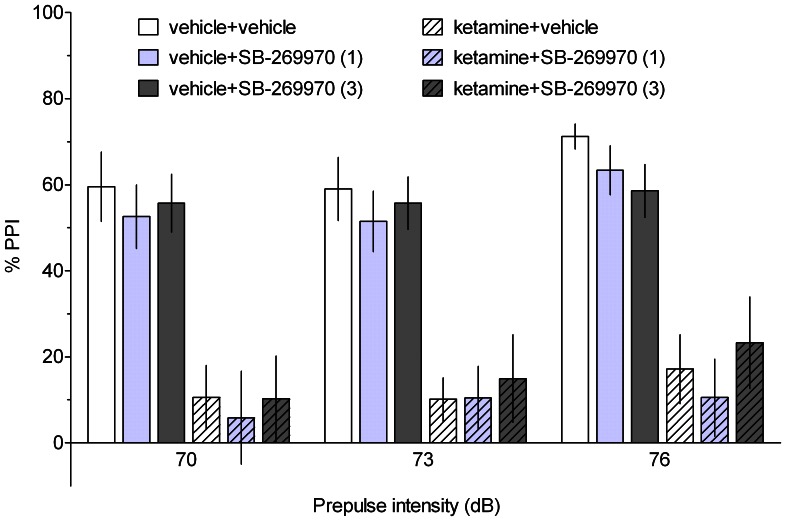
The effect of SB-269970 on the ketamine-induced disruption of PPI. PPI was evaluated at three prepulse intensities (70, 73 and 76 dB). Data are shown as the mean ± S.E.M. N = 6–8 rats per group.

**Figure 7:**
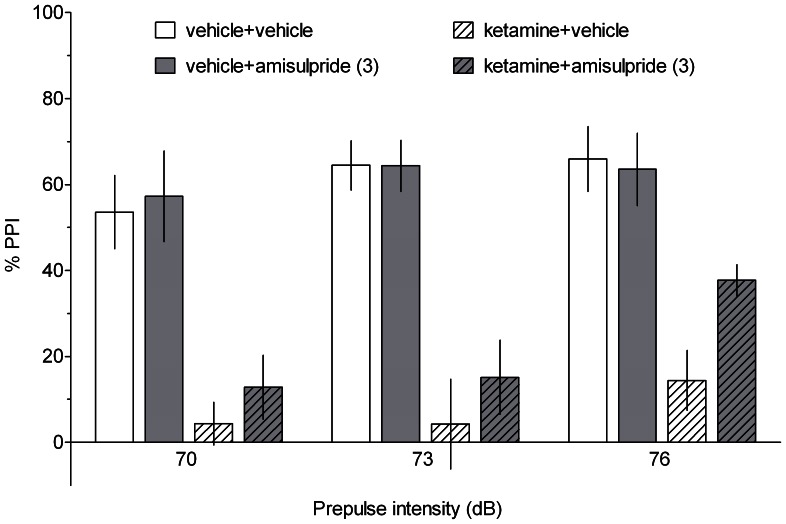
The effect of amisulpride on the ketamine-induced disruption of PPI. PPI was evaluated at three prepulse intensities (70, 73 and 76 dB). Data are shown as the mean ± S.E.M. N = 6–10 rats per group.

As illustrated in Figures 6 and 7, ketamine significantly reduced PPI. No dose of SB-269970 or amisulpride significantly altered ketamine-induced disruption. Three-way ANOVAs revealed significant effects of ketamine treatment (SB-269970 study: F[1], [37] = 67.65, p<0.001 and amisulpride study: F[1], [30] = 63.21, p<0.001) and prepulse intensity (SB-269970 study: F[2,74] = 4.67, p<0.05 and amisulpride study: F[2,60] = 4.41, p<0.05). There were no significant effects of SB-269970 or amisulpride treatment and no significant two- or three-way interactions.

**Figure 8:**
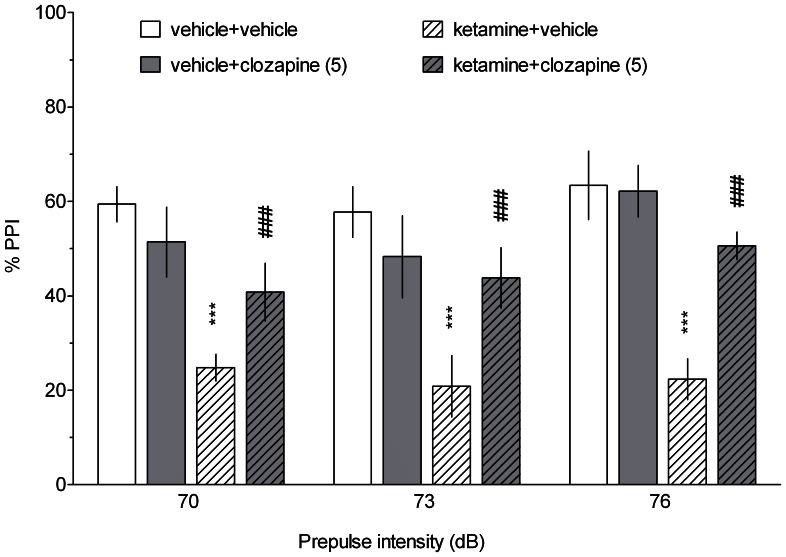
The effect of clozapine on the ketamine-induced disruption of PPI. PPI was evaluated at three prepulse intensities (70, 73 and 76 dB). Data are shown as the mean ± S.E.M. N = 7–10 rats per group. Symbols: ***p<0.001 significant reduction in PPI compared to the vehicle-treated animals, ^##^p<0.01 significant improvement in PPI compared to the ketamine-treated animals (post-hoc analysis of a significant ketamine x clozapine interaction).

In contrast, the ketamine-evoked deficit was ameliorated by clozapine; significant differences between ketamine+vehicle- and ketamine+clozapine-treated groups were noted irrespective of prepulse intensities (Figure 8). A three-way ANOVA of %PPI revealed a significant effect of ketamine treatment (F[1], [26] = 22.75, p<0.001), prepulse intensity (F[2,52] = 3.68, p<0.05) and a significant interaction of ketamine and clozapine treatment (F[1], [26] = 8.63, p<0.01). There were no other significant two- or three-way interactions.

Drug effects on startle amplitude are demonstrated in Table 3. Two-way ANOVAs revealed no significant interactions of ketamine with SB-269970 (F[2], [37] = 0.46, NS), amisulpride (F[1], [30] = 2.95, NS) or clozapine (F[1], [26] = 0.28, NS) treatments.

**Table 3:** Effects of SB-269970, amisulpride and clozapine on startle amplitude in the vehicle- and ketamine-treated rats.

Treatment (mg/kg)	Amplitude		
vehicle+vehicle	519	±	68
vehicle+SB-269970 (1)	718	±	124
vehicle+SB-269970 (3)	688	±	140
ketamine+vehicle	687	±	116
ketamine+SB-269970 (1)	686	±	103
ketamine+SB-269970 (3)	803	±	99
vehicle+vehicle	754	±	143
vehicle+amisulpride (3)	1402	±	262
ketamine+vehicle	792	±	109
ketamine+amisulpride (3)	882	±	162
vehicle+vehicle	988	±	203
vehicle+ clozapine (5)	828	±	186
ketamine+vehicle	832	±	166
ketamine+ clozapine (5)	872	±	204

Results represent the mean ± S.E.M. baseline (pulse alone) startle amplitude.

## Discussion

The present study revealed that acute administration of the selective 5-HT7 receptor antagonist, SB-269970, and amisulpride ameliorated ketamine-induced cognitive inflexibility and novel object recognition deficit in rats. Both compounds were also effective in attenuating the ketamine-evoked disruption of social interactions. In contrast, neither SB-269970 nor amisulpride affected the ketamine-disrupted prepulse inhibition. The ketamine-induced reduction in ultrasonic vocalisations was also not reversed by either treatment.

SB-269970-induced inhibition of the NOR deficit agrees with previous preclinical data demonstrating its efficacy (at a similar dose-range as used in the current study) in ameliorating recognition memory impairment induced by subchronic PCP administration [7]. The beneficial action of SB-269970 in the ASST, to our knowledge, is the first demonstration of the efficacy of the selective blockade of 5-HT7 receptors against schizophrenia-like cognitive inflexibility in the NMDAR antagonist-based model. The action of SB-269970 on set-shifting and object recognition in ketamine-treated animals also corroborates previous findings demonstrating its effectiveness in other cognitive domains. Accordingly, SB-269970 attenuated the PCP-induced deficits in reversal learning [9] as well as dizocilpine-induced impairment in a delayed non-matching to position task in rats [6]. SB-269970 also reversed memory deficits demonstrated in an autoshaping Pavlovian instrumental learning task in rats after systemic administration of dizocilpine [28] or after intra-PFC infusion of ketamine [29]. Moreover, another antagonist of 5-HT7 receptors, SB-656104-A, reversed dizocilpine-induced learning and memory impairments in the passive avoidance and Morris water maze tests in rats [8]. These data, together with the current findings, support the procognitive action of 5-HT7 antagonists in animal models of schizophrenia.

While the present and previous findings indicate the effectiveness of 5-HT7 receptor antagonism in overcoming schizophrenia-like cognitive impairments, the role of these receptors in modulating cognitive processes under physiological conditions remains equivocal. It should be noted that 5-HT7 receptor inactivation has been shown to disrupt hippocampal-dependent learning and memory functions as assessed in the novel location, contextual fear conditioning and Barnes maze tests [30], [31]. Specifically, the deficits associated with 5-HT7 receptor blockade appear to be related to particular aspects of memory, i.e., the hippocampus-based allocentric representation [30], [31]. Nevertheless, with regard to the cognitive tasks used in the present experiments, both 5-HT7 knock-out and SB-269970-treated mice performed comparably to control animals in the NOR task [31]. In the present NOR experiment, neither SB-269970 nor amisulpride affected performance of cognitively unimpaired control animals. It is not surprising, as the applied experimental setting (a 1-h ITI) did not allow for the demonstration of potential procognitive activity. Nevertheless, it cannot be excluded that the investigated drugs can ameliorate the delay-induced impairment of recognition memory. Indeed, using a 24-h ITI, Waters et al. [32] demonstrated SB-269970-induced improvement in recognition memory, albeit at a much higher dose (30 mg/kg) than used in the present experiments.

Moreover, according to our previous study [33], SB-269970 facilitated set-shifting performance in cognitively unimpaired control rats. Amisulpride produced a similar, though less pronounced effect. These results suggest that the procognitive action of the 5-HT7 receptors antagonism may not be restricted to the schizophrenia-like state induced by the NMDAR blockade.

Since the attentional set-shifting is a food-rewarded task, drug-induced changes in the motivation for food could potentially affect rats’ performance. However, there were no differences in the mean time to collect the food reward during the ED phase between treatment groups in either SB-269970 or amisulpride study (data not shown). Thus, it seems unlikely that the present results could be explained by the effects on animal’s appetite.

SB-269970 is characterised by a relatively short half-life [34]. Nevertheless, it has been demonstrated that the compound was able to enhance catecholamine levels in the rat mPFC up to 160 min post-injection [35]. Therefore, it is likely that SB-269970 still affects rats' performance at the ED phase of the ASST, which occurs approximately 140 min after drug administration.

Although the precise pathway by which the selective blockade of 5-HT7 receptors ameliorates ketamine-induced cognitive deficits is unknown, experimental data suggest some neurochemical mechanisms that could underlie this action. For example, Bonaventure et al. [6] demonstrated that the effectiveness of SB-269970 in ameliorating dizocilpine-induced cognitive deficits was accompanied by normalisation of the dizocilpine-evoked enhancement of glutamate efflux in the rat mPFC. It has been suggested that the increased glutamate release in the mPFC, a secondary effect of NMDAR blockade, may be responsible for the NMDAR antagonist-induced cognitive impairment [36]. Thus, the beneficial action of SB-269970 against cognitive deficits in the NMDAR hypofunction model may be explained by a reduction of the enhanced glutamate release. An alternative, but not mutually exclusive, hypothesis involves increased prefrontal catecholamine efflux as the mechanism underlying the putative cognitive enhancements in schizophrenia. Accordingly, the increase in mPFC noradrenaline and dopamine levels produced by SB-269970 [35] might explain the beneficial profile of this compound in the current study. In line with this explanation, our previous study demonstrated that the dopamine and noradrenaline reuptake inhibitor, mazindol, reversed the ketamine-induced deficits in rats' set-shifting performance [25].

The next finding of the current study was that amisulpride, an antipsychotic drug with a high affinity for 5-HT7 receptors, demonstrated procognitive effects similar to those of the selective 5-HT7 receptor antagonist, SB-2669970. The role of 5-HT7 receptor antagonism in the procognitive action of amisulpride was supported by the study of Horiguchi et al. [7]. The authors demonstrated that the ability of amisulpride (3 mg/kg) to reverse PCP-induced deficits in the NOR task was blocked by co-treatment with the 5-HT7 receptor agonist AS 19. Moreover, using 5-HT7 knock-out mice, Abbas et al. [10] confirmed the contribution of 5-HT7 antagonism to the mechanisms underlying the antidepressant action of amisulpride; the drug effect in the tail suspension test required functional 5-HT7 receptors. Nevertheless, other possible mechanisms underlying the procognitive action of amisulpride cannot be excluded. For example, the dopamine D3 receptor antagonistic properties of amisulpride may also participate in its beneficial action. In fact, a D3 receptor antagonist evoked procognitive effects in animal models of schizophrenia [37]. It should be noted, however, that sulpiride, an antipsychotic with a significantly lower affinity for 5-HT7 receptors than amisulpride but with similar D2/D3 antagonistic properties, failed to reverse the PCP-induced NOR impairment [7].

In contrast to the beneficial action of SB-269970 and amisulpride in cognitive tasks, these compounds did not affect ketamine-induced disruption of prepulse inhibition. The lack of efficacy of 5-HT7 antagonists agrees with previously reported data demonstrating that SB-269970 did not reverse the deficits in PPI evoked by ketamine in rats [38] or by PCP in rats and mice [39]. However, it should be noted that 5-HT7 receptors may play a partial role in the glutamatergic PPI model, as 5-HT7 knock-out mice were less prone to the PCP-induced disruption of PPI than wild-type mice [39]. In addition, another 5-HT7 receptor antagonist, SB-258741, normalised PCP-disrupted PPI [22]. While we cannot exclude the possibility that higher doses of SB-269970 could be effective in this study, the compound at 10–30 mg/kg was also ineffective in the NMDAR antagonist-based PPI models [38], [39]. In addition, it cannot be excluded that amisulpride administered at a higher dose would have reversed the ketamine-induced impairment. However, it is likely that amisulpride, being devoid of significant affinity for 5-HT2A receptors (K_i_  =  8.3 microM; [10]), would be ineffective in the glutamatergic PPI models. Indeed, it has been suggested that antagonism at 5-HT2A receptors plays a major role in ameliorating the disruption of PPI induced by NMDAR antagonists [40]. Of note were the positive effects of clozapine, an antipsychotic drug with an affinity for 5-HT2A receptors (Figure 8).

The present study further demonstrated that SB-269970 and amisulpride ameliorated ketamine-induced deficits of social interaction. To the best of our knowledge, this is the first demonstration of the effectiveness of these compounds in the NMDAR antagonist-based model of social withdrawal. Another antagonist of 5-HT7 receptors, SB-258741, had no beneficial effect on PCP-disrupted SI [22]. This discrepancy may be explained by the different properties of SB-269970 and SB-258741 [41] as well as a number of differences in the experimental protocol, including the strain of rats (Wistar vs. Sprague-Dawley), the applied NMDAR antagonist (PCP vs. ketamine), the scoring method (automated vs. manual) and drug administration schedules (3 days vs. 1 day). While one cannot exclude the possibility that the pro-social action of the 5-HT7 antagonist may also reflect an anxiolytic-like action of the tested compounds [42], the present experimental conditions (dim light, intense adaptation) were set to maximise social behaviour in control rats. Thus, a purported anxiolytic-like action (i.e., a further increase in time spent in social interactions) would not be expected. Further, neither SB-269970 nor amisulpride affected the time spent in social interaction when administered alone (data not shown). While the drug-induced alteration in exploratory activity may potentially affect the SI measure, the applied drug treatments did not affect distance traveled, as revealed in the acquisition trial of the NOR task. Thus, it seems unlikely that the outcome of the SI test may reflect the drug-induced changes in exploratory activity. It is worth noting that among the several antipsychotics tested, only a few of them (remoxipride and aripiprazole) were able to reverse deficits in social behaviour induced by NMDAR blockade [43], [44].

The present results are also the first demonstration that the ability of SB-269970 and amisulpride to reduce social withdrawal due to NMDAR blockade was not accompanied by a similar effect on ultrasonic vocalisations. The 50 kHz “happy” calls have been postulated as an index of the rat’s positive emotions akin to human joy [45], and they are reduced by dizocilpine, albeit unaffected by ligands of opioid, dopamine, serotonin and cholinergic receptors [46]. These observations could suggest that only some aspects of ketamine-induced social deficits are normalised by the antipsychotics and that clearly more research is needed to further elucidate this objective measure of social withdrawal. Likewise, neurochemical substrates regulating emission of 50 kHz USVs are still poorly understood. In this regard of note is the amisulpride- and SB-269970-evoked decrease of 50-kHz calls in vehicle-treated rats. Similar effect was previously demonstrated for clozapine, risperidone and the D2 receptor antagonist, pimozide [47]. However, while the latter study suggested the role of D2 receptors in producing 50-kHz USVs, the potential involvement of 5-HT7 receptors remains unknown.

In summary, the present study demonstrated the effectiveness of the 5-HT7 antagonists SB-269970 and amisulpride in ameliorating ketamine-induced cognitive inflexibility, recognition memory impairment and social withdrawal. It is likely that antagonism of 5-HT7 receptors represents a useful pharmacological approach in the treatment of cognitive deficits and negative symptoms of schizophrenia.
